# Meta‐analysis of whole‐brain radiotherapy plus temozolomide compared with whole‐brain radiotherapy for the treatment of brain metastases from non‐small‐cell lung cancer

**DOI:** 10.1002/cam4.1306

**Published:** 2018-03-07

**Authors:** Yong Xin, WenWen Guo, Chun Sheng Yang, Qian Huang, Pei Zhang, Long Zhen Zhang, Guan Jiang

**Affiliations:** ^1^ Department of Radiotherapy Affiliated Hospital of Xuzhou Medical University Xuzhou, Jiangsu 221002 China; ^2^ Department of Dermatology Affiliated Huai'an Hospital of Xuzhou Medical University Huai'an Jiangsu 223002 China; ^3^ Department of Dermatology Affiliated Hospital of Xuzhou Medical University Xuzhou Jiangsu 221002 China

**Keywords:** brain metastases, NSCLC, radiotherapy, TMZ

## Abstract

The aim of this meta‐analysis was to compare the efficiency of whole‐brain radiotherapy (WBRT) plus temozolomide (TMZ) with WBRT for the treatment of brain metastases from non‐small‐cell lung cancer (NSCLC). For dichotomous variables, outcomes were reported as relative risk ratio (RR) and 95% confidence interval (CI) was used to investigate the following outcome measures: overall response rate, headache, gastrointestinal adverse reactions, and hematological adverse reactions. Twelve randomized controlled trials involving 925 participants (480 received WBRT plus TMZ; 445 received WBRT) were included in the meta‐analysis. There was a significant difference between the overall response rate (RR = 1.40, 95% CI 1.24–1.57; *Z* = 5.51; *P* < 0.00001), gastrointestinal adverse reactions (RR = 1.46, 95% CI 1.05–2.04; *Z* = 2.27; *P* = 0.02), and hematological adverse reactions (RR = 1.45, 95% CI 1.04–2.02; *Z* = 2.21; *P* = 0.03) of patients treated with WBRT plus TMZ compared with patients treated with WBRT alone. There was no significant difference between headaches (RR = 1.11, 95% CI 0.93–1.02; *Z* = 1.13; *P* = 0.26) in patients treated with WBRT plus TMZ compared with patients treated with WBRT alone. In conclusion, the currently available evidence shows that WBRT plus TMZ increases the overall response rate in patients with brain metastases of NSCLC compared with WBRT alone.

## Introduction

Lung cancer is the most common malignant tumor worldwide; 85% of which is pathologically diagnosed as non–small‐cell lung cancer (NSCLC) 1. The 5‐year survival rate of NSCLC is very low. Approximately, 15–30% of NSCLC patients develop brain metastases 1 with symptoms including headache, vomiting, and visual disturbances. The median survival time of untreated patients with brain metastases is <3–6 months 2. The incidence of brain metastases has significantly increased because of the use of better diagnostic methods and the improvement of public health awareness. The main treatments for patients with NSCLC are surgery, radiotherapy, and chemotherapy 3. However, the efficacy of these traditional treatments in brain metastases is poor. Therefore, there is an urgent need to develop new strategies for the treatment of brain metastases from NSCLC.

Temozolomide (TMZ) is a second‐generation alkylating agent widely used for treating brain malignant tumors. TMZ enters the central nervous system (CNS) easily, and its plasma concentrations are increased easily, enhancing the antitumor activity 4, 5, 6. The main adverse reactions include nausea, vomiting, fatigue, and hematologic responses, which can be controlled with some drugs. TMZ combined with radiation therapy can be used to treat newly diagnosed pleomorphic glioblastoma tumors or recurrent malignant gliomas. Patients have good tolerance of TMZ and it achieves good clinical efficacy, improving patient quality of life.

At present, a few randomized controlled trials (RCTs) have shown that TMZ combined with whole‐brain radiotherapy (WBRT) can significantly improve the remission rate of brain metastases of NSCLC. In addition, TMZ can enhance the radiosensitizing effect of WBRT 7. As these RCTs only analyzed a relatively small number of patients and most were retrospective studies, large‐scale data are needed to confirm the effectiveness of this combination therapy. The aim of this meta‐analysis was to compare the efficiency and adverse reactions of WBRT plus TMZ with WBRT alone for the treatment of brain metastases from NSCLC.

## Materials and Methods

### Data sources

We searched the Wanfang Database, PubMed, the Cochrane Library, Medline, and Elton B. Stephens Company (EBSCO) using the keywords “temozolomide OR TMZ” AND “radiotherapy” AND “brain metastases” AND “non‐small‐cell lung cancer OR NSCLC.” The publication dates were from 1 January 2002 to 1 June 2017 with no restrictions in language.

### Study selection

The inclusion criteria for the RCTs were: (i) pathologically confirmed NSCLC and diagnosed brain metastases with computed tomography (CT) or magnetic resonance imaging (MRI); (ii) aged over 18 years; (iii) radiotherapy and chemotherapy tolerance; (iv) expected lifetime of more than 3 months; (v) compared WBRT versus WBRT plus TMZ; and (vi) reported sufficient data on outcomes.

Exclusion criteria were: (i) nonrandomized and nonclinical controlled trials; (ii) trials with missing data; and (iii) duplicate reports, trials of poor methodological quality, and trials with obvious bias.

The authors carefully analyzed the methodology of the articles to select the qualified RCTs. With the keywords used, 91 papers (67 in Chinese and 24 in English) were identified. According to the inclusion and exclusion criteria, we independently examined the full text and discussed each article together.

### Data extraction and synthesis

The investigators carefully extracted data from the eligible studies. Data included the name of the first author, year of publication, journal name, quality of the study, intervention, number of patients in the study, dosage and duration of the two groups, median survival time, and the number of patients with adverse reactions.

### Main outcome(s) and measure(s)

The following outcomes were measured: overall response rate, headache, gastrointestinal adverse reactions, and hematologic adverse reactions. Overall response was defined as complete response and partial response which was measured by brain CT or MRI according to World Health Organization criteria. Toxicity was evaluated according to the National Cancer Institute Common Terminology Criteria for Adverse Events version 3.0. The number of patients with adverse reactions was collected directly from the published papers. Disagreement regarding data extraction was resolved by discussion and consensus among the investigators.

### Quality assessment

The authors used the “Cochrane handbook for systematic reviews of interventions version 8 5.0.0” to assess the methodological quality of the included RCTs, which assessed: (i) generation of the random allocation scheme (random sequence generation); (ii) allocation concealment; (iii) blinding of participants and personnel; (iv) blinding of outcome assessment; (v) incomplete outcome data; (vi) selective reporting; and (vii) other sources of bias.

### Statistical methods

The data were analyzed using Review Manager v.5.3 software (Cochrane Collaboration, Oxford, UK) and SPSS 16.0. For dichotomous variables, outcomes were reported as relative risk ratio (RR) and 95% confidence interval (CI). A *P*‐value of <0.05 was considered statistically significant. The heterogeneity test with inconsistency index (*I* ^2^) statistic and *Q* statistic was performed 9. If the outcomes were found to have good homogeneity (*P* > 0.1; *I* ^2^ ≤ 50%), a fixed effect model was used for secondary analysis; if not (*P* < 0.1; *I* ^2^ > 50%), a random‐effect model was used 9.

## Results

**Figure 1:**
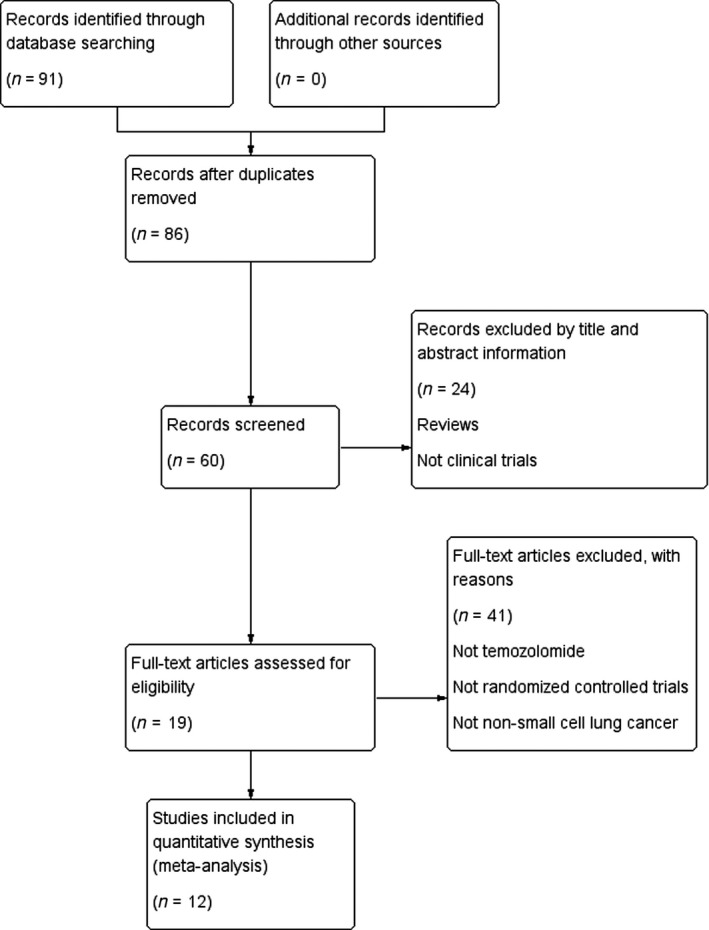
Flowchart of article screening and selection process.

As mentioned above, we identified the eligible paper carefully. The literature selection process is presented in the PRISMA flow chart (Fig. 1) according to the PRISMA guidelines. After comprehensive discussion and analysis, 12 RCTs were selected and included for final meta‐analysis.

### Included studies

**Figure 2:**
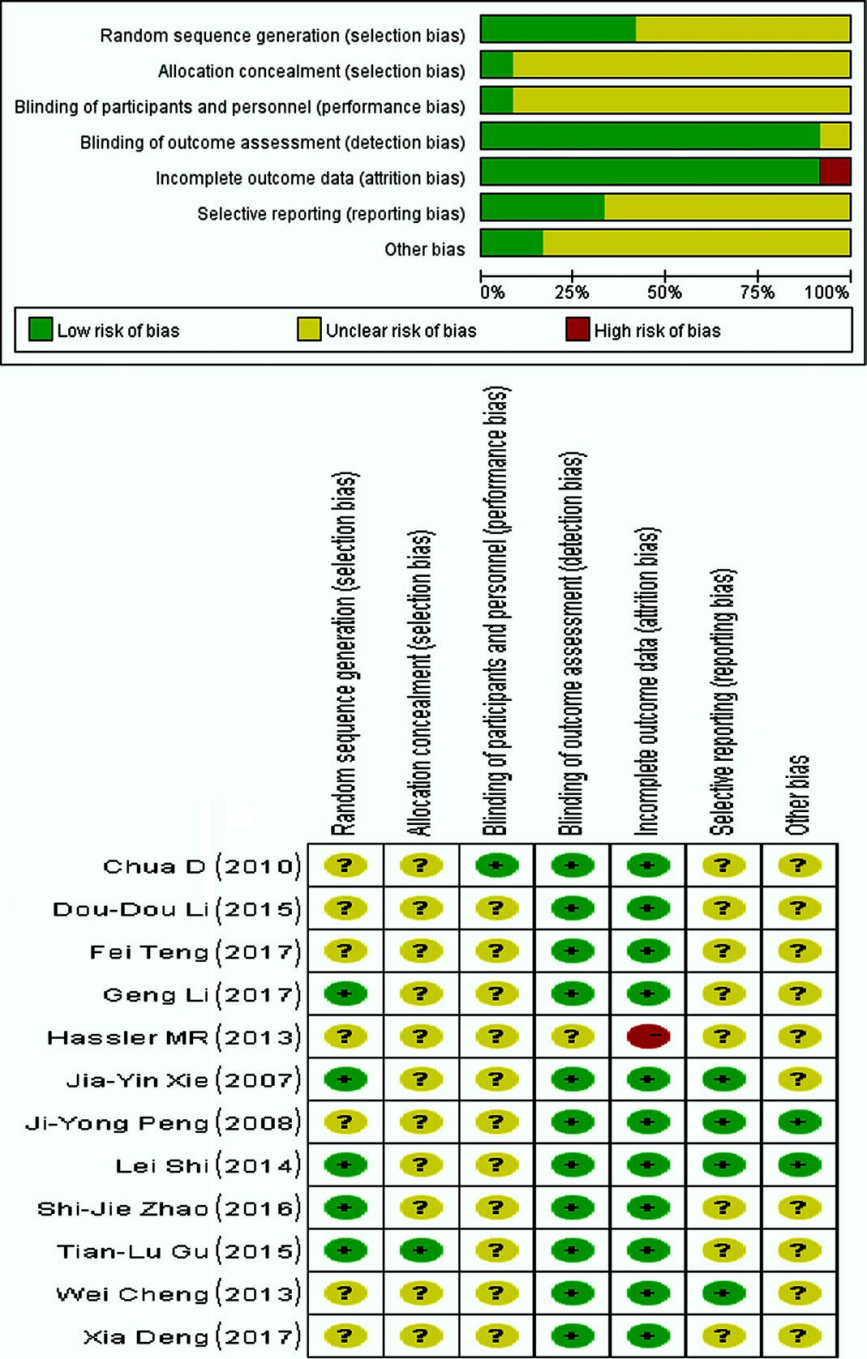
Summary diagram of risk of bias percentile chart.

**Figure 3:**
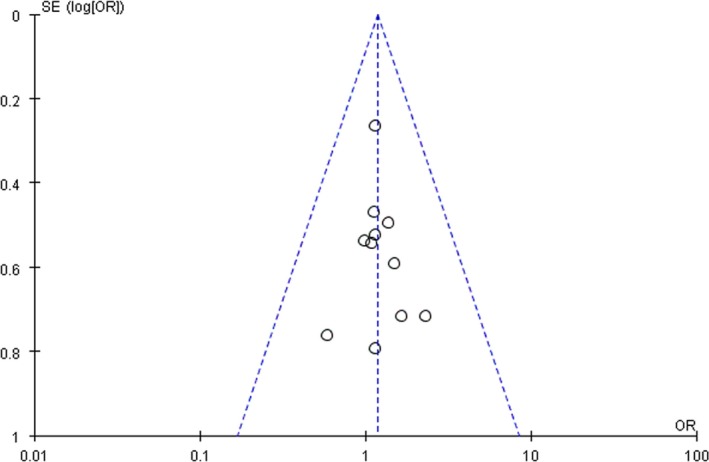
Funnel plot of publication bias.

In total, 925 patients were enrolled in the 12 included RCTs. The patients all had pathologically confirmed NSCLC and brain metastases diagnosed with CT or MRI. Of the 925 patients, 480 received WBRT plus TMZ and 445 received WBRT. Radiation and drug dose varied in different studies. The characteristics of the 12 included RCTs are shown in Table 1. Figure 2 depicts the assessment of the methodological quality of the 12 RCTs. The funnel plot, which was highly symmetrical, was used to analyze the publication bias (Fig. 3).

**Table 1:** Summary of the characteristics of the 12 trials included in the meta‐analysis (1 cycle = 28 days)

Author	Groups	No of patients (WBRT + TMZ/WBRT)	Treatment	Gender (Male/Female)	Age (Average age OR Median age)	Pathological type (Squamous cell carcinoma/Adenocarcinoma/Other)	References
Chua, et al. (2010)	WBRT + TMZ	47	WBRT (30 Gy/10 f) + TMZ (75 mg/m^2^ * 21 d or 28 d)	30/17	38–78 (59)	‐	6
	WBRT	48	WBRT (30 Gy/10 f)	32/16	43–79 (62)		
Hassler, et al. (2013)	WBRT + TMZ	22	WBRT (30 Gy/10 f or 40 Gy/20 f) + TMZ (75 mg/m^2^ * 2 weeks + 100 mg/m^2^*14 d*6 cycles)	13/9	36–85 (69)	‐	16
	WBRT	13	WBRT (30 Gy/10 f or 40 Gy/20 f)	8/5	54–78 (64)		
Yong Peng, et al. (2008)	WBRT + TMZ	19	WBRT (30 Gy/10 f or 40 Gy/20 f) + TMZ (200 mg/m^2^*5 d * 3 cycles)	11/8	35–71 (54)	‐	23
	WBRT	21	WBRT (30 Gy/10 f or 40 Gy/20 f)	14/7	32–72 (52)		
Shi, et al. (2014)	WBRT + TMZ	43	WBRT (40 Gy/20 f) + TMZ (75 mg/m^2^ during WBRT)	25/18	38–72 (55)	12/29/2	21]
	WBRT	41	WBRT (40 Gy/20 f)	24/17	38–73 (56)	27/12/2	
Cheng, et al. (2013)	WBRT + TMZ	30	WBRT (40 Gy/20 f) + TMZ (200 mg/m^2 ^* 5 d * 6 cycles)	18/12	39–70 (52)	‐	19
	WBRT	26	WBRT (40 Gy/20 f)	11/5	38–71 (54)		
Xie, et al. (2007)	WBRT + TMZ	25	WBRT (40 Gy/20 f) + TMZ (200 mg/m^2 ^* 5 d)	36/14	30–70 (56)	20/28/2	[18]
	WBRT	25	WBRT (40 Gy/20 f)				
Fei, et al. (2017)	WBRT + TMZ	26	WBRT (30 Gy/10 f or 40 Gy/20 f) + TMZ (75 mg/m^2^ during WBRT)	16/10	‐	14/9/3	24
	WBRT	25	WBRT (30 Gy/10 f or 40 Gy/20 f)	14/11		13/8/4	
Li, et al. (2017)	WBRT + TMZ	39	WBRT (40 Gy/20 f) + TMZ (75 mg/m^2^*4 weeks +150 mg/m^2^*5 d * 6 cycles)	19/20	21–70 (47.37 ± 4.56)	‐	22
	WBRT	39	WBRT (40 Gy/20 f)	21/18			
Tian Lu (2015)	WBRT + TMZ	52	WBRT (40 Gy/20 f) + TMZ (200 mg/m^2^ * 5 d * 4 cycles)	28/24	46–65 (58.9 ± 5.9)	47/55/0	26
	WBRT	50	WBRT (40 Gy/20 f)	23/27			
Doudou, et al. (2015)	WBRT + TMZ	18	WBRT (40 Gy/20 f) + TMZ (75 mg/m^2^*4 weeks + 100 mg/m^2^ * 5 d * 6 cycles)	10/8	39–70 (54.5)	‐	7
	WBRT	18	WBRT (40 Gy/20 f)	11/7	37–72 (53.5)		
Zhao (2016)	WBRT + TMZ	30	WBRT (40 Gy/20 f) + TMZ (75 mg/m^2^*4 weeks + 150 mg/m^2 ^* 5 d * 6 cycles)	16/14	38–69 (58.1 ± 3.3)	‐	13
	WBRT	30	WBRT (40 Gy/20 f)	17/13	36–68 (57.6 ± 3.3)		
Deng (2017)	WBRT + TMZ	129	WBRT (30 Gy/10 f)+TMZ (75 mg/m^2^ during WBRT + 100 mg/m^2 ^* 5 d * 6 cycles)	69/60	34–85 (60)	‐/227/‐	25
	WBRT	109	WBRT (30 Gy/10 f)	67/42			

WBRT, whole‐brain radiotherapy; TMZ, temozolomide.

### Overall response rate

**Figure 4:**
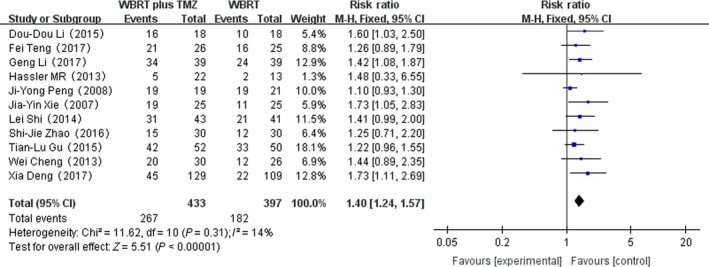
Forest plot of overall response rate in whole‐brain radiotherapy (WBRT) plus TMZ compared with WBRT.

Eleven RCTs that reported the overall response rate were selected in the analysis. The heterogeneity test for overall response rate was not statistically significant, allowing the data for each outcome to be calculated using the fixed effects model (*I* ^2^ = 14%; *P* = 0.31). The meta‐analysis indicated that there was a significant difference in the overall response rate in patients treated with WBRT plus TMZ compared with WBRT alone (RR = 1.40, 95% CI 1.24–1.57; *Z* = 5.51; *P* < 0.00001) (Fig. 4).

### Median survival time

According to SPSS analysis, there was a significant difference about median survival time in patients between the two groups in relevant 10 RCTs (*P* < 0.05) (Table 2). And the median survival time of the WBRT plus TMZ was longer than that of the WBRT alone.

**Table 2:** Median survival time in trials included in the meta‐analysis (*P < 0.05)

	Chua (2010)	Hassler (2013)	Yong Peng (2008)	Shi, et al. (2014)	Cheng, et al. (2013)	Xie, et al. (2007)	Fei, et al. (2017)	Li, et al. (2017)	Tian Lu. (2015)	Doudou, et al. (2015)	Zhao (2016)	Deng (2017)
WBRT + TMZ*	4.7	8.6	13	10.56	12.8	8.6	–	–	8.4	6.0	10.67	8.5
WBRT	4.3	7.0	11	6.24	8.2	4.5			7.8	4.9	6.18	5.9

WBRT, whole‐brain radiotherapy; TMZ, temozolomide.

### Headache

**Figure 5:**
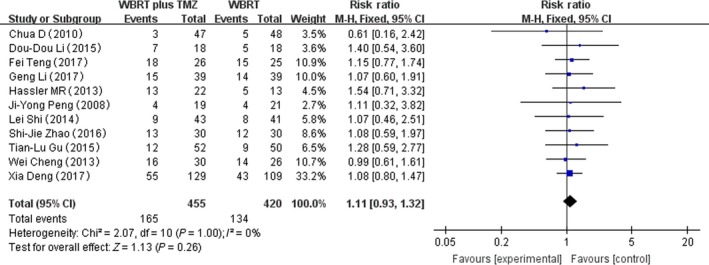
Forest plot of headache in whole‐brain radiotherapy (WBRT) plus TMZ compared with WBRT.

Eleven RCTs that reported headache adverse reactions were selected in the analysis. The heterogeneity test for headache was not statistically significant, allowing the data for each outcome to be calculated using fixed effects models (*I* ^2^ = 0%; *P* = 1.00). The meta‐analysis indicated that there was no significant difference in the headache adverse reactions in patients treated with WBRT plus TMZ compared with WBRT alone (RR = 1.11, 95% CI 0.93–1.02; *Z* = 1.13; *P* = 0.26) (Fig. 5).

### Gastrointestinal adverse reactions

**Figure 6:**
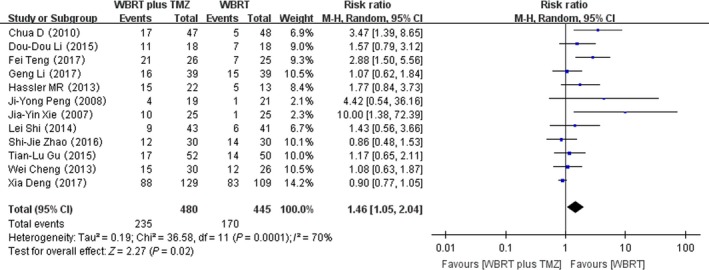
Forest plot of gastrointestinal adverse reactions in whole‐brain radiotherapy (WBRT) plus TMZ compared with WBRT.

Twelve RCTs that reported gastrointestinal adverse reactions were selected in the analysis. The heterogeneity test for gastrointestinal adverse reactions was statistically significant, allowing the data for each outcome to be calculated using random effects models (*I* ^2^ = 70%; *P* = 0.0001). The meta‐analysis indicated that there was a significant difference in the gastrointestinal adverse reactions in patients treated with WBRT plus TMZ compared with WBRT alone (RR = 1.46, 95% CI 1.05–2.04; *Z* = 2.27; *P* = 0.02) (Fig. 6).

### Hematologic adverse reactions

**Figure 7:**
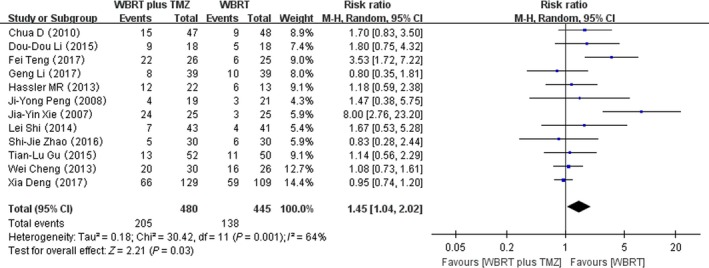
Forest plot of hematologic adverse reactions in whole‐brain radiotherapy (WBRT) plus TMZ compared with WBRT.

Twelve RCTs reporting on hematologic adverse reactions were selected in the analysis. The heterogeneity test for hematologic adverse reactions was statistically significant, allowing the data for each outcome to be calculated using random effects models (*I* ^2^ = 64%; *P* = 0.001). The meta‐analysis indicated that there was a significant difference in the hematologic adverse reactions in patients treated with WBRT plus TMZ compared with WBRT alone (RR = 1.45, 95% CI 1.04–2.02; *Z* = 2.21; *P* = 0.03) (Fig. 7).

## Discussion

In this meta‐analysis, we compared WBRT plus TMZ with WBRT for the treatment of brain metastases of NSCLC. The meta‐analysis proved that there was a significant difference in the overall response rate in patients between WBRT plus TMZ and WBRT alone. Our findings indicate that the combination therapy can significantly increase the overall response rate, prolong the median survival time, and enhance the antitumor effect.

Approximately, 15–30% of NSCLC patients develop brain metastases 1, which presents a particular challenge. In the past, the standard therapy for brain metastases was WBRT 10, which may provide local disease control, but only has a marginal survival benefit 6. Unfortunately, owing to the local disease control by WBRT, there is still a high risk in both CNS and systemic progression. The median survival time of patients having WBRT is only 3–4 months after the diagnosis of the brain metastases 6. Therefore, radiotherapy is often combined with chemotherapy for patients with brain metastases 11, 12.

WBRT can prolong survival, but most patients survive less than half a year after radiation. It is difficult for many chemotherapeutic agents to reach the focus through the blood–brain barrier, leading to poor efficacy 13. TMZ is a second‐generation, alkylating agent with antitumor activity in the brain tumor 14, 15. The role of chemotherapy for treating brain metastases from NSCLC remains controversial because of its chemical toxicity 16. Chemotherapy drugs can cross the blood–brain barrier, leading to high concentrations in the CNS. Some past experiments indicate that the efficacy of TMZ is enhanced by radiotherapy 7, 16. However, the cytotoxic effect of TMZ might be enhanced by its radiosensitization during WBRT in brain metastases. Evidence concerning the efficacy of chemotherapy combined with radiotherapy in patients with brain metastases is accumulating 17. TMZ can act on various stages of tumor cell division and ultimately promote apoptosis 18, 19.

The important outcome of cancer treatment is overall response rate. According to Figure 4, there was a significant difference in the overall response rate in patients treated with WBRT plus TMZ compared with WBRT alone. However, each RCT reported different radiotherapy doses and TMZ dosages. The overall response rate in the combination therapy group was still 1.4 times greater than that in the WBRT group, which indicates the positive effect of the combination therapy 20. Another important endpoint in oncology research is survival time. However, as each of the included studies did not provide sufficient data, we could not analyze the median survival time using the hazard ratio (HR). From the published articles, we extracted the relevant data for median survival time of the two comparison groups from 10 RCTs (Table 2). Our findings indicate that the combination therapy prolongs the median survival relative to WBRT alone (Table 1). The mechanism of the antitumor activity may be: (1) Chemotherapy drugs can control the development of the primary lesion and metastatic brain lesions; (2) WBRT disrupts the blood–brain barrier function, rendering it easier for TMZ to cross the blood–brain barrier, thereby improving the curative effect 21, 22. Although there were more adverse effects in the combined therapy group, they were resolved using medication 23, 24, 25. The patients did not discontinue the treatment because of the TMZ adverse effects. TMZ mainly causes nausea, vomiting, and fatigue, which most patients can tolerate 7. These results all prove that WBRT combined with TMZ is effective and safe.

The meta‐analysis had several limitations. As shown in Table 1, the meta‐analysis still analyzed only a limited number of eligible studies and a relatively small number of patients. Of the nine Chinese‐language RCTs and the three English‐language RCTs, only four demonstrated random allocation methods such as the envelope method and the random number table, while the remaining studies did not specify the method of randomization. In addition, 11 RCTs did not mention the blinding of participants and personnel (Fig. 2). Although the general characteristics of the patients, such as age and Karnofsky performance score (KPS), were roughly the same, the results still involved selection bias. Moreover, as positive results are more likely to be published, publication bias should also be taken into consideration. However, the standard of outcomes including overall response rate, headache, gastrointestinal adverse reactions, and hematologic adverse reactions are objective; therefore, the lack of blinding of observers would not have caused significant bias.

In conclusion, the currently available evidence shows that WBRT plus TMZ can increase the overall response rate in patients with brain metastases of NSCLC, compared with WBRT alone. TMZ can cause some adverse reactions, which are mostly self‐limiting and can be controlled with drugs. Large, high‐quality, double‐blind trials are needed to confirm the efficiency of WBRT plus TMZ 26. The optimal mode and dose of radiotherapy and chemotherapy also needs further research.

## Conflict of Interest

The authors declare that they have no competing interests.

## Declaration

Ethics approval and consent to participate: Not applicable; Consent for publication: Not applicable; Availability of data and material: All data generated or analyzed during this study are included in published article.
